# Supplementary material for: All-fiber frequency agile triple-frequency comb light source
Source: Nat Commun. 2023 Dec 1;14:7953. doi: 10.1038/s41467-023-43734-w (PMC10692102; doi:10.1038/s41467-023-43734-w)
Supplement: Supplementary file 1 — Supplementary Information [file 41467_2023_43734_MOESM1_ESM.pdf]

Supplementary Information : All-fiber frequency agile triple-frequency comb light source

Eve-Line Bancel[1,2], Etienne Genier [1], Rosa Santagata[2], Matteo Conforti[1], Alexandre Kudlinski[1], Géraud Bouwmans[1], Olivier Vanvcincq[1], Damien Labat[1], Andy Cassez[1], and Arnaud Mussot[1*]
[1] *Univ. Lille, CNRS, UMR 8523 - PhLAM - Physique des Lasers Atomes et Molécules, F-59000 Lille, France and*
[2]*ONERA, 91120 Palaiseau, France*

Detailed experimental set-up – Fig. S1 (a) represents the optical set-up. An ultra-narrow CW laser (100 Hz, Koheras) centered at 1550 nm delivering 40 mW is amplified up to 500 mW before being split into three channels. Channels 1 and 2 are almost similar. AOMs are inserted in channels 1 and 2 to shift the carrier envelop offset frequency to 100 MHz and 200 MHz respectively. Then the CW laser is intensity modulated (iXBlue MXER-LN) to generate a pulse train of 55 ps pulse duration whose repetition rate can be tuned from 100 MHz to 10 GHz in our system (most of the experiments had been performed at 0.5 GHz). The signal is then amplified and the amplified stimulated emission (ASE) in excess is removed by using a spectral filter. A second intensity modulator is used to increase the extinction ratio between the pulses to reach about 50 dB. This allows for increasing the peak power of the pulses after they get amplified in the EDFA, and removes the central component in the spectrum, corresponding to the CW background between the pulses. In Channel 3, there is a single modulator and we clearly see a significant CW component. In all channels, we get about 600 mW input average power (which is equivalent to 21 W of peak power at f_{rep} = 500 MHz with 55 ps pulse duration). The light beam is injected within the tri-core fiber by using a commercial FAN (Chiralphotonics) with about 1 dB splice loss. The fiber is 1 km long, the linear loss is 1 dB/km, the nonlinear coefficient is 5 /W/km and the dispersion is 5 ps^2/km. These characteristics are almost similar in each core. At the fiber output, the frequency combs are demultiplexed by the mean of a FAN with about 2.5 dB splice loss for each core. The three combs are available at the output of three SMF 28 fibers. The system is PM (polarization maintaining) till the FAN at the input of the tri-core fiber.

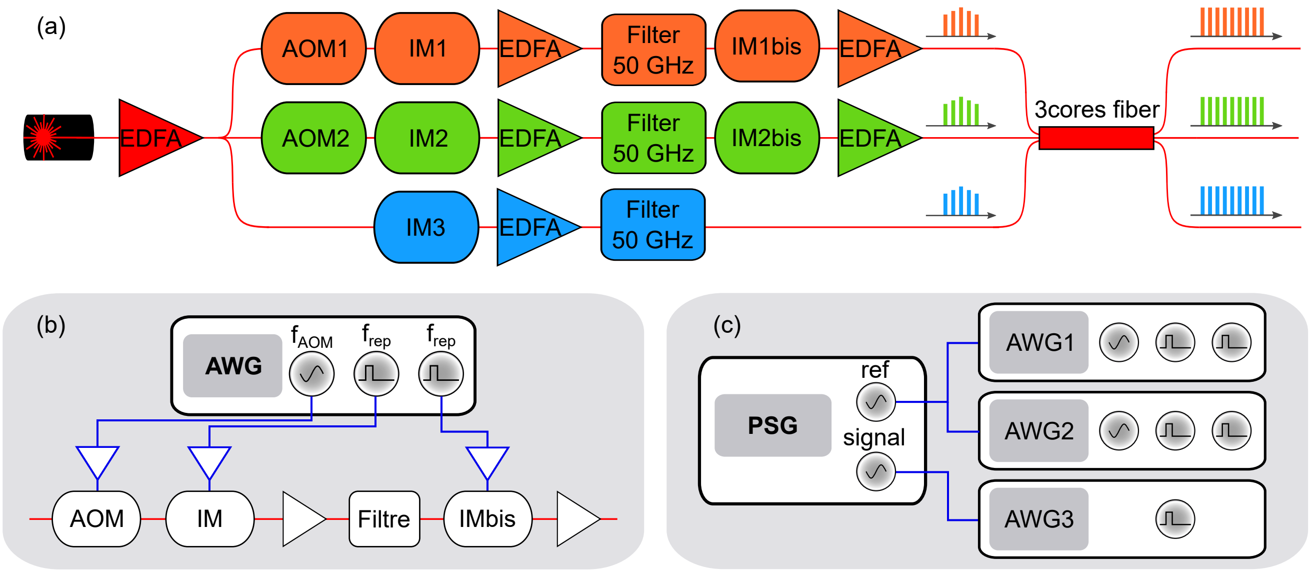

FIG. S1. **Detailed optical and RF setup (a) Scheme of the optical setup. (b) RF driver scheme for one channel.** For CH1 and CH2, an AWG drives the AOM and the two IMs. For CH3, the AWG drives only one IM. **(c) Global RF driver scheme.** The three AWGs are connected to a common PSG, used as a reference for two channels, and as a clock for the third channel. EDFA = erbium-doped fiber amplifier; ASE = amplified spontaneous emission; AWG = arbitrary waveform generator; PSG = power signal generator.

* arnaud.mussot@univ-lille.fr

RF pulse generation – On each channel, one AWG drives all the RF inputs (IMs and optional AOM), as presented in Fig.S1 (b). Using AWGs to generate RF pulses gives the advantages of tunability and ease of use. However, the sampling rate is limited (25GHz / 50GHz depending on the model) and its resolution (+/- 100 MHz) does not allow much freedom on the difference of repetition frequencies δf_{rep}. To overcome this limitation, we use a Power Signal Generator (PSG) - Keysight E8257D (Fig.S1 (c)). Two of the AWGs share its 10 MHz reference. The signal output of this PSG is used as a clock for the third AWG in order to finely tune its repetition rate. The PSG signal frequency $f_{clock,PSG}$ is slightly shifted compared to the actual AWG clock frequency $f_{clock,AWG}$, which leads to the actual repetition rate f_{rep}' = f_{rep} + δf_{rep} to be slightly shifted compared to the theoretical repetition rate: δf_{rep} = f_{rep} x (1 - $f_{clock,PSG}/f_{clock,AWG}$). This is a way to ensure that the three AWGs share a common reference while being able to tune the repetition rate as freely as possible. The inputs are commutable and the repetition rate can be independently changed on each channel.

Self-Phase Modulation –In Fig.S4 (a) we recorded the evolution of the spectrum at the output of core 1 as a function of the input average power with an optical spectrum analyzer. At 19 mW, the spectrum is almost similar to the input spectrum (see in Fig.1 (d) of the main article). As the power increases, the spectrum broadens due to SPM [1]. With P_{in} = 650 mW, we achieve a spectral broadening of 7 nm or almost 1 THz at 1550 nm which corresponds to the spectra depicted in Fig. 1 of the main article. There is a slight peak at the center that is a residue of the CW pump. The spectrum is symmetrical and flat-top, with a SNR higher than 20 dB. Fig.S4 (b) represents the corresponding numerical simulations by integrating the Nonlinear Schrodinger equation (NLSE) [1]. We used experimental parameters which are listed in Fig.S4's caption. Note that we considered the limited extinction ratio of the IM in these numerics with 50 dB. Thus, we confirm through these numerics the origin of the central component is indeed due to the CW background between the pulses due to the limited extinction ratio of the modulators.

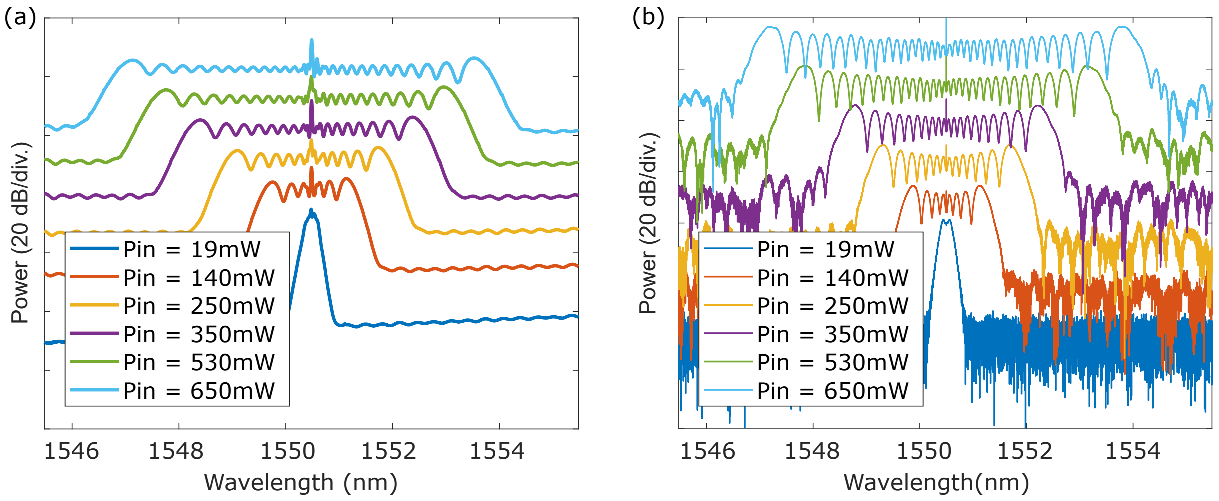

FIG. S2. **Evolution of the spectral broadening as a function of the input power**. (a) Experimental recording. Example of Comb 3. (b) Numerical simulations from the NLSE. Parameters: γ = 5 $W^{-1}.km-1$, β_2 =5 ps^2/km, and α = 1 dB/km at 1550 nm, extinction ratioof the IM is 50 dB and Gaussian pulses of 55 ps duration FWHM. The average input power varies from 19 mW to 650 mW.

Temporal compression – We demonstrated the output combs can be efficiently compressed. We measured the chirp experimentally as follows. We use an ultra-narrow tunable pass-band filter (EXFO XTM50 - 50 GHz FWHM) to record the delay of each spectral component as a function of the central one. We used an ultra-fast oscilloscope combined with a high band-pass photo-detector (70 GHz bandpass each). We then integrated this curve to calculate the phase curve depicted in Fig.S7 (a). We then used a commercial spatial light modulator (WaveShaper (WS) - Coherent 1000A) to imply the reversed-phase law to compress the pulses. Fig.S7 (b) presents the auto-correlation (AC) trace we experimentally recorded (purple) after compression. It has a duration of 2 ps at FWHM. In comparison, for reference, we calculated the AC trace of the FT of the experimental spectrum (green curve in Fig.S7 (b)). The AC trace duration is 1.3 ps at FWHM which corresponds to a pulse duration of 650 fs. Since the pulses have no standard shapes, we have to deduce the FWHM duration of the experimental trace from this ratio (factor 2 between the AC trace and the pulse duration). We can then conclude the FWHM duration of the recompressed pulses is 1 ps. The recompression is almost perfect, the slight discrepancy is due to the limited spectral resolution of the Waveshaper (10 GHz) which avoids reproducing the slight phase modulation on the bottom of the curve (green curve in Fig.S7 (a)).

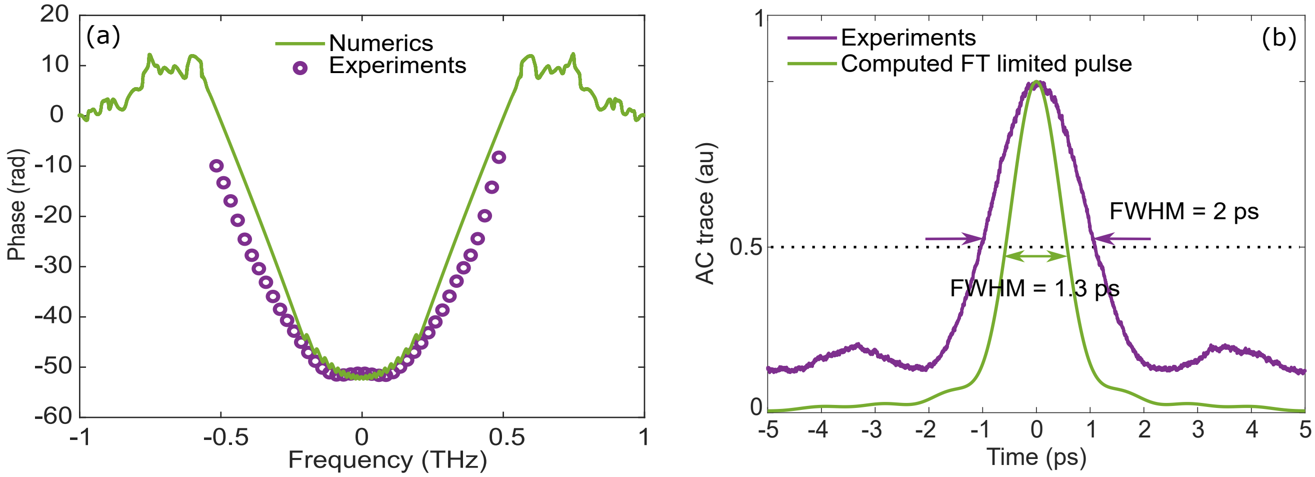

FIG. S3. **Temporal compression of the pulses** at the output of CH2. **(a) Spectral phase** measured (purple circles), and by numerical simulation (green line). **(b) AC trace** of one pulse after compression. AC = auto-correlation. WS = WaveShaper. FWHM = full width at half maximum

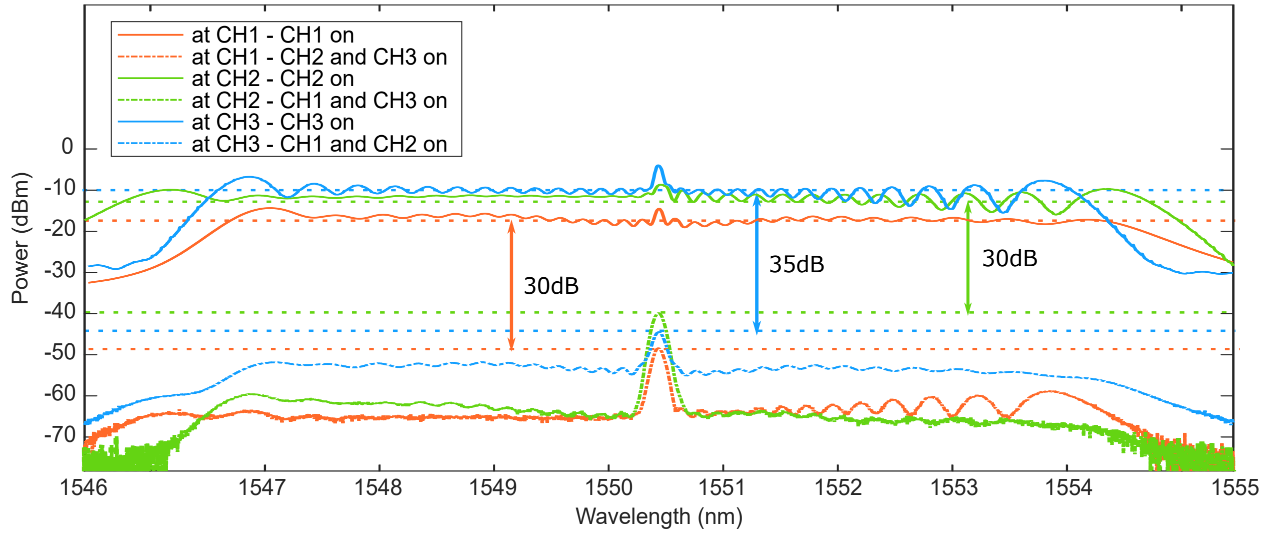

FIG. S4. **Cross-talk between the three combs.** OSA spectra of the output of the tri-core fiber for Comb1 (orange), Comb2 (green) and Comb3 (blue). The full lines correspond to the output of one core when two other ones are off. The dotted lines correspond to the output of one core when the two other ones are on.

Cross-talk between the three channels – We estimated the cross-talk between the different cores as follows. We measured the power collected at the output of one core when the other two cores are on compared to the case when it is on. The pairs of spectra are repaired by identical colors in Fig.S4. At minimum, a difference of more than 30 dB, had been observed even 35 dB for core 3. From these measurements, we can consider as negligible the cross-talks between the cores.

Phase noise of the three combs – The first two rows of Fig.S5 present the phase noise of the first line for each comb at two different repetition rate. The similarity between the curves indicates that the noise types are the same for all three combs. One noticeable difference is the rising of the photo-detection floor with the increase in the number of lines. This applies when passing from the laser (blue curve) to the EO comb (orange curve), to the broaden comb (purple comb), since the spectra are broadened at each stage, and the measurements are made at constant mean power on the photo-detector. It is also true when the repetition rate goes from 1.25 GHz (first row) to 500 MHz (second row), meaning more spectral elements in the bandwidth of the photo-detector (1.6 GHz), still at constant mean power. The laser measurement (blue curve) indicates the reference of the phase noise for the chosen experimental conditions.

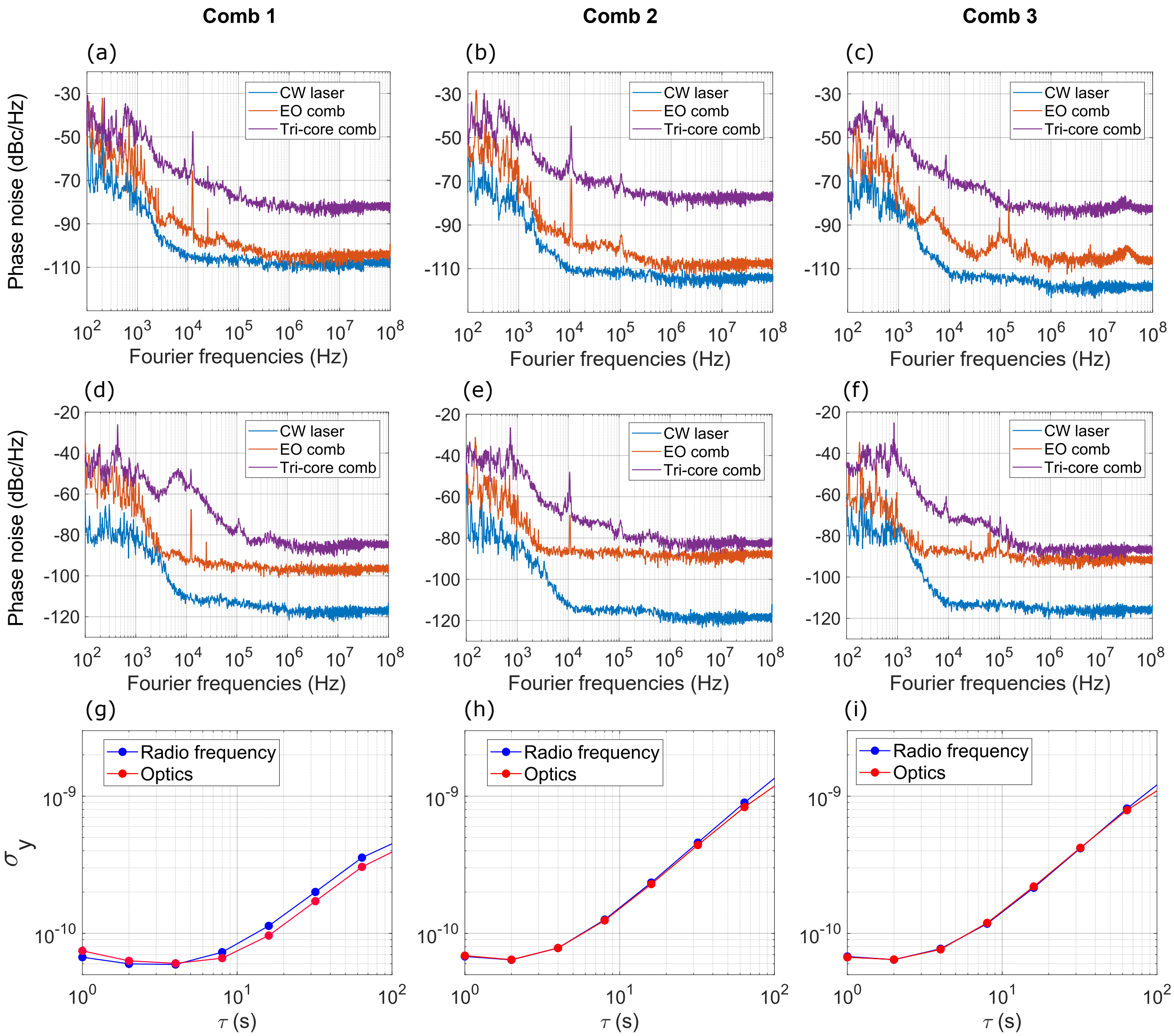

FIG. S5. **First two rows (a)-(f): SSB phase noise of the fundamental spectral component** of the three combs at different stages of the set-up for two different repetition rates: $f_{rep} = 1.25$ GHz ((a) (b) and (c)) and $f_{rep} = 500$ MHz ((d) (e) and (f)). The different curves (blue) indicate the accumulated phase noise of the laser after frequency shifting (blue), after EOMs (orange) and after broadening (purple). **Third row (g)-(i): Allan deviation of the repetition rate for the three combs** for the RF signals driving the EOMs (blue) and the optical signals (red).

Allan deviation of the three combs – The third row of Fig.S5 presents the Allan deviation of the RF and optical repetition rates for each comb. The relative frequencies have been computed compared to the repetition rate $f_{\rm rep} = 500$ MHz. The same shapes of curves, between the RF and the optical subsytems, as well as between the three combs are featured. There is a small gap between the measurement for Comb 1 and the two other combs. This affects the value of σ_y at $\tau = 100$ s for instance, but not the minimum stability floor at $\sigma_y = 6.10^{-11}$.

Dual-comb RF spectra between each pair of combs – Fig.S6 presents the RF spectra from the interferometry between (a) comb1 and comb3 and (b) comb2 and comb3 , at $f_{\rm rep} = 500$ MHz and $\delta f_{\rm rep} = 50$ kHz. Fig.S6 (c) and (d) present a zoom on the peaks. The spectra are calculated over n = 10 interferograms, averaged N = 20000 times for (a) and (b), over n = 10 interferograms, averaged N = 2000 times for (c) and (d). The preponderance of the central peak in is due to the poorer extinction of the continuous background for comb3.

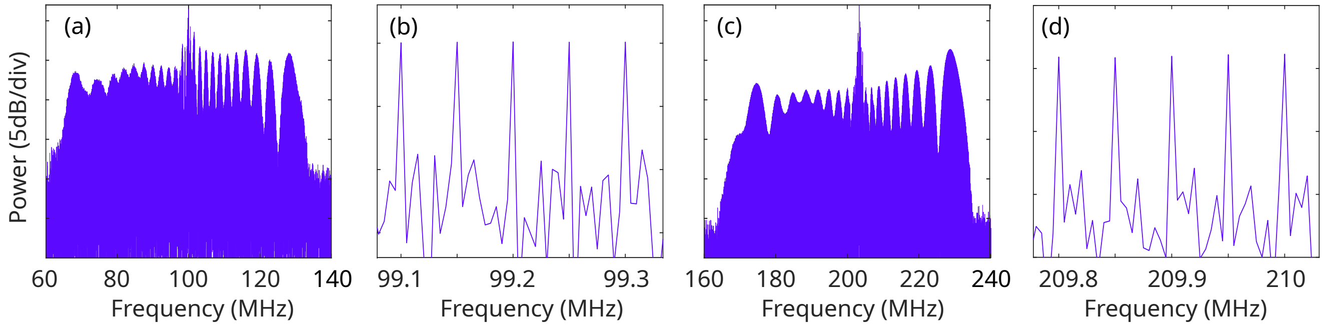

FIG. S6. **Dual-comb RF spectra of the three combs** at $f_{\rm rep} = 500$ MHz and $\delta f_{\rm rep} = 50$ kHz (a) between comb1 and comb2, (b) zoom on the peaks, (c) and between comb1 and comb3, (d) zoom on the peaks.

FWM and tri-comb interferometry – Fig.S7 RF spectrum resulting from the FWM between two combs when the delay is tuned and analyzed by a third comb which pays the role of a local oscillator. Fig.S7-(a) corresponds to Fig. 4 (d) of the main paper, reproduced for the sake of comparison. Fig.S7-(b) correspond to numerical simulations using almost experimental parameters. To reduce the computational time, we neglect the linear loss of the fiber and fairly assume SPM is the dominant effect during the propagation in the fiber. That way, we can calculate the spectra at the tri-core fiber output by simply considering the nonlinear operator. We then calculated the FWM between two combs by integrating the NLSE in a highly nonlinear fiber (peak power 5 W, L=300 m, $\beta_2 = 1 \times 10^{-28}$ ps^2/km and $\gamma = 10/W/km$) and calculated the interferogram with a third comb. For a large pump-probe delay, relative to the pulse duration (55 ps), no FWM component is generated, while we observe a clear FWM signal when the two pumps temporally overlap. The spectrogram shows intensity modulations due to those of the pumps and LO originating from SPM effect. A slight chirp is also visible due to the fact the three combs are not Fourier transform limited as they originate from SPM. The numerical results are in fairly good agreement with the experiments.

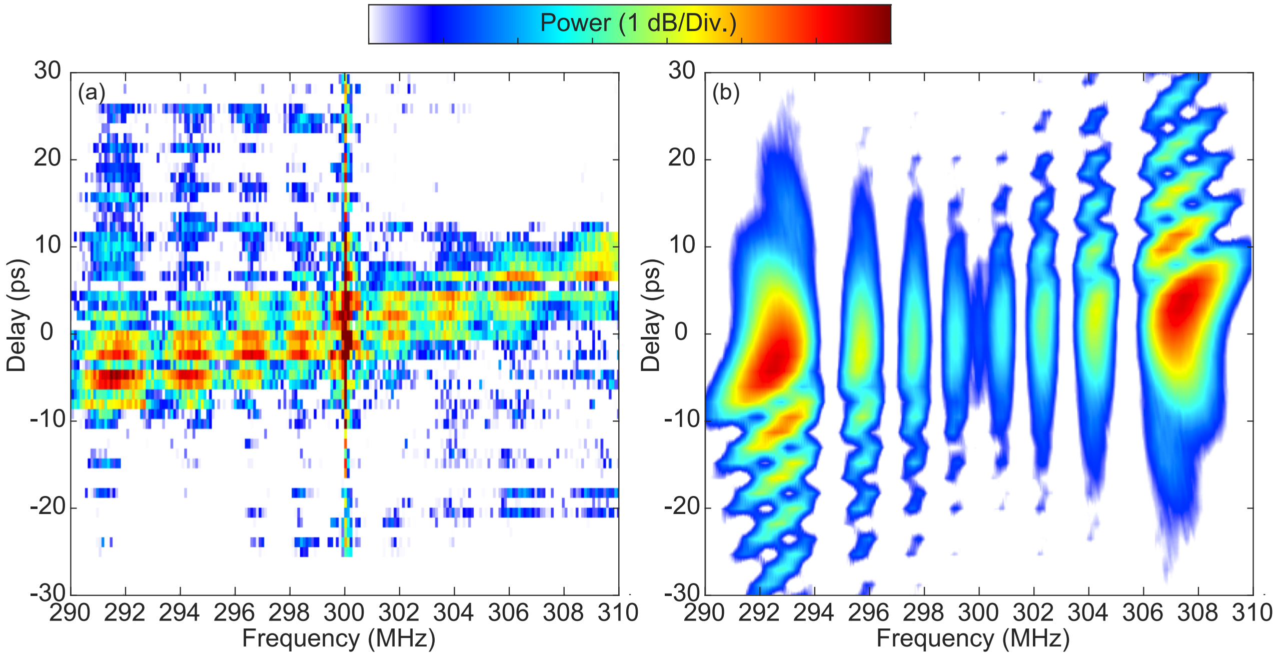

FIG. S7. Envelop of the spectrogram of the FWM component centred at 300 MHz. (a) Experimental results from Fig. 4 (d) of the main text. (b) Numerical simulations.

Supplementary References

[1] G. P. Agrawal, in Nonlinear Fiber Optics (Elsevier, 2013).
